# Predictors of gambling and problem gambling in Victoria, Australia

**DOI:** 10.1371/journal.pone.0209277

**Published:** 2019-01-23

**Authors:** Piers D. L. Howe, Adriana Vargas-Sáenz, Carol A. Hulbert, Jennifer M. Boldero

**Affiliations:** Melbourne School of Psychological Sciences, University of Melbourne, Melbourne, Victoria, Australia; Tampereen Yliopisto, FINLAND

## Abstract

In 2016, the gambling habits of a sample of 3361 adults in the state of Victoria, Australia, were surveyed. It was found that a number of factors that were highly correlated with self-reported gambling frequency and gambling problems were not significant predictors of gambling frequency and problem gambling. The major predictors of gambling frequency were the degree to which family members and peers were perceived to gamble, self-reported approval of gambling, the frequency of discussing gambling offline, and the participant’s Canadian Problem Gambling Severity Index (PGSI) score. Age was a significant predictor of gambling frequency for certain types of gambling (e.g. buying lottery tickets). Approximately 91% of the explainable variance in the participant’s PGSI score could be explained by just five predictors: *Positive Urgency; Frequency of playing poker machines at pubs*, *hotels or sporting clubs; Participation in online discussions of betting on gaming tables at casinos; Frequency of gambling on the internet*, and *Overestimating the chances of winning*. Based on these findings, suggestions are made as to how gambling-related harm can be reduced.

## Introduction

Gambling is a common pastime and is found in all countries in the world, although in some, such as the United Arab Emirates, Brunei and Cambodia, it is either illegal or highly restricted. For most individuals, their gambling does not cause problems but for some individuals their gambling harms either themselves, their family or their friends. This harm can be financial, emotional or social. Worldwide, the standardized past year rate of problem gambling varies from a low of 0.12%-0.5% to a high of 5.8%-7.6%, depending on the specific country and the exact scale used to assess it [1, 2]. In general, the rates of problem gambling are lowest in Europe, intermediate in North America and Australia and highest in Asia. The present study focussed on the state of Victoria, Australia, as a condition of its funding.

Gambling is widespread in Victoria, Australia. In 2015–2016, Victorians spent AU$5.79 billion on gambling, spending AU$5.02 billion on gaming machines, AU$494 million on race betting and AU$281 on sports betting [3]. According to a large-scale survey of Victorians conducted in 2014, in the previous 12 months, about 70% of the population had gambled [4]. In particular, 47% of Victorians had bought lottery tickets; 42% had bought tickets in raffles, sweeps and other competitions; 21% had betted on horse and dog racing; 11% had bet using scratch tickets; 17% had placed bets on gaming machines; 6% had participated in phone-based betting competition; 5% had bet on casino table games, and 5% had bet on sports. In the state of Victoria, the rate of problem gambling is estimated to lie in the range of 0.4%-0.8% [4, 5].

As reviewed below, previous studies have shown that a large number of factors are correlated with both an individual’s personal gambling frequency and gambling problems. However, different factors were evaluated in different studies, making it hard to determine their relative importance. Furthermore, it was not clear how many of these factors significantly predict gambling frequency and gambling problems. The aim of the present study was to simultaneously evaluate a large number of the factors that are known to be associated with gambling, to determine their *relative* importance as predictors of gambling frequency and gambling problems. Only by knowing which factors are important predictors of gambling frequency and problem gambling can effective behavioural interventions be devised.

### Factors associated with gambling and problem gambling

Problem gambling occurs when a person’s gambling harms either themselves or other people. For the purposes of our study, we have operationalized problem gambling using the 9-item Canadian Problem Gambling Severity Index (PGSI) scale [6]. This scale asks a series of questions to determine the extent to which a person’s gambling is likely to be problematic or causing harm. If, based on their responses to this questionnaire, it is determined that an individual’s gambling is likely to be causing harm, they would be classified as a problem gambler. The full scale and scoring details are reproduced in the Appendix. The sections below review the current literature on the factors that are known to correlated with an individual’s gambling frequency and the probability that they have gambling problems. For convenience, related factors have been grouped together.

We must acknowledge from the outset that there are more potential predictors of gambling frequency and problem gambling that could be investigated in our study. We note in particular the reviews by Dowling et al. [7] and Hing, Russel, Tolchard and Nower [8]. Like Hing et al., we chose to include predictors of age, gender, language at home and type of gambling. We also chose to include a number of other factors, for the reasons detailed below. In particular, instead of asking about household composition we asked about the actions and beliefs of family, friends and others. The review by Dowling et al. was not available when we designed our study, so it did not influence our study design.

#### Perceived gambling frequency, approval of gambling by others and discussing gambling

It is known that an individual’s gambling frequency and the probability that they have gambling problems are correlated with not just the degree to which they personally approve of gambling [9], but also with the degree to which they believe others gamble and approve of gambling [10–15]. For example, a study by Moore and Ohtsuka [13] of adult Australians found that the degree to which a person’s family and friends gamble and approve of gambling predicted an individual’s gambling frequency. In addition, due to the availability heuristic [16], it is likely that the more an individual discusses gambling with fellow gamblers the more he/she is likely to perceive others to gamble and to approve of gambling. Thus, the frequency of discussing gambling is likely to be correlated with an individual’s gambling frequency and probability of having gambling problems.

#### Exposure to advertising

Gambling is often portrayed inaccurately in advertisements [17, 18]. In particular, advertisements typically attempt to make gambling appear glamorous and do not mention the negative aspects of gambling. Deans, Thomas, Derevenksy, and Daube [19] reported their participants felt that the sheer volume of sports betting advertisements normalised sports betting and effectively encouraged them to bet on sports (see also [20] and [21]). Consistent with this, exposure to gambling advertisements has been repeatedly found to increase the frequency of gambling by adolescents and young adults [22–24]. Governments and government agencies periodically attempt to counter these pro-gambling messages. For example, the Victorian Responsible Gambling Foundation is currently promoting its *Love the game*, *not the odds* initiative, which is a social media campaign designed to disrupt the normalisation of gambling in sport. However, it is hard for such social media campaigns to counter-frame the overwhelmingly pro-gambling message [25]. For example, moderate risk and problem gamblers in Victoria reported that messages about risk and help seeking were “drowned out” by advertisements promoting gambling [26]. Similarly, Lee, Lemanski, and Jun [27] found that while media campaigns that emphasized the problems associated with gambling did reduce gambling intentions, pro-gambling media campaigns were more effective at increasing intentions to gamble.

#### Demographic factors

Gambling participation and problem gambling are generally greatest for individuals in their 20s and 30s [28–30]. Gender is also known to influence gambling, with males being more likely than females to gamble [24, 28, 29, 31–36]. Additionally, males report more gambling problems than females [17, 24, 37–43] and are more likely to be problem gamblers [41, 44–46]. Finally, studies that have differentiated between skill-based (e.g. poker, card games) or chance-based (e.g., bingo, lotto) activities have found that males prefer skill-based ones whereas females prefer chanced-based ones [47]; though Aasved [48] found that males and females are equally likely to play poker machines in both casinos and other venues. Females are more likely to participate in scratch tickets, bingo, phone/SMS competitions, and in raffles/sweeps [49].

Additional demographic factors associated with an increased likelihood of problem gambling in Australia are being born outside Australia, speaking a language other than English at home, and residing in metropolitan areas [31]. Finally, problem gambling often results in separation and divorce [50–52] (for a review see [53]). For example, in the study by Holdsworth et al., of the eighteen couples interviewed where one partner had a gambling problem, eight were separated and attributed their separation at least in part to gambling, specifically to the loss in trust caused by their partner’s gambling.

#### Psychological factors

More frequent gambling is related to depression [37, 54, 55] and, compared to those who are not problem gamblers, problem gamblers report higher levels of depression [41], but see [46]. Similarly, self-esteem tends to be lower in individuals who are problem gamblers than those who are not [56]. Indeed, it is thought that low levels of self-esteem result in increased gambling [57]. Sensation seeking and impulsivity are also associated with more frequent gambling [58] and gambling problems [37, 59]. Cyders and Smith [60] found that the tendency to act rashly when in a positive mood (i.e., positive urgency) was associated with longitudinal increases in students’ gambling behaviour during the freshman year, whereas the tendency to act rashly when upset (i.e., negative urgency) was not.

Problem gamblers believe that they are “luckier” than non-problem gamblers [37] and more frequent gambling amongst students is associated with having more inaccurate or erroneous gambling cognitions [61], such as believing that one can influence gambling outcomes. Steenbergh, Meyers, May, and Whelan [62] found that overestimating one’s chances of winning at gambling, a factor they labelled luck/perseverance, and having illusions of control over gambling outcomes differentiated college students and community members who gambled from those who did not. However, they also reported that these factors did not distinguish between pathological and problem gamblers (i.e., those who gamble compulsively and those who experience problems as a result of their gambling; [63]).

#### Aim of study

From the above literature review, it was predicted that the following factors would be correlated with an individual’s self-reported gambling frequency and PGSI score: the degree to which others are perceived to gamble and approve of gambling, the degree to which individuals discuss gambling, the degree to which individuals see advertisements and receive promotional material, their age, gender, country of birth, language spoken at home, location (metropolitan versus rural), and relationship status. It is possible that the predictors of gambling frequency would differ from those of problem gambling, at least in their relative importance [64]. The purpose of the current study was to determine the relative importance of the above predictors in determining both gambling frequency and problem gambling. Only by knowing the relative importance of these various predictors can future research focus on the more important predictors.

## Method

Participants were sourced from an online survey panel, operated by The Online Research Unit (ORU). The ORU is an Australian research company and is certified by the International Organization for Standardization (ISO 20252 and ISO 26362). The ORU maintains a panel of volunteers who have agreed to participate in online surveys. A mix of incentives including vouchers and charitable donations of small value is provided to participants via the ORU in return for participating in these surveys. Crucially, the participants in our study had an ongoing relationship with the ORU and understood that their responses would be completely anonymous. This was important as this would have encouraged them to disclose any gambling problems they may have had, as gambling problems are stigmatized [8] and it is known that people are more likely to reveal sensitive information when guaranteed that their responses will be anonymous, as opposed to merely being confidential [65]. The ORU invited members of their survey panel to participate in the online survey via email. To avoid biasing the recruitment, this email did not specify the nature of the survey (i.e., it did not mention that it was related to gambling). In recruiting participants, the ORU matched for age, sex, and location (metropolitan vs regional) relative to the general Victorian population as determined by the demographic data supplied by the 2011 Australian Bureau of Statistics (ABS) survey. (The data for the 2016 census had not yet been released when the survey was conducted). This ensured that the sample was as representative as possible. However, because these participants were drawn from a study panel whose members were self-selected, this sample is not necessarily representative of the general population. The exact breakdown of the sample relative to age, gender and location is detailed in the supplementary information. The analysis was conducted using IBM SPSS version 22 [66].

### Ethical approval

The study received ethical approval from the University of Melbourne Human Research Ethics Committee (ID: 1545085). All participants gave written consent to participate in this study.

### Measures

The full survey is included in the supplementary information. It was conducted online and is briefly summarised here.

#### Demographic characteristics

Participants were asked to provide information about their age, gender, country of birth, relationship status, main language spoken at home, and residential postcode.

#### Gambling frequency

Participants were asked to indicate to what extent they had participated in 12 commonly-available gambling activities during the past year. In addition, they were asked to what extent they believed that their family, peers, and people in general had participated in each of these 12 activities in the past year. So as to be comparable with previous work, the same gambling activities were surveyed and the questions were phrased in the same way as previously [32]. In particular, respondents were asked to indicate whether, in the past year, they had gambled more than six times, less than six times or not at all.

#### Approval of gambling

Participants were asked to indicate the extent to which they approve of each of the 12 gambling activities surveyed. They also indicated the extents to which they believed that their family, their peers, and general population separately approve of gambling on these 12 gambling activities. For each question, they responded on a five-item scale ranging from “Strongly Disapprove” to “Strongly Approve”.

#### Advertisements, promotional materials, and gambling-related discussions

Participants were asked to indicate how frequently they had seen advertisements for each of the 12 gambling activities and also how frequently they had received promotional material. Participants were also asked how frequently they discussed the gambling activities either online (e.g., internet discussion boards) or offline (e.g., in person). As before, for each question, participants were offered three choices, more than six times in the last year, less than six times in the last year, or never.

#### Canadian problem gambling severity index (PGSI) scale

The nine-item Canadian Problem Gambling Severity Index (PGSI) was used to assess the extent to which an individual’s gambling causes problems [6]. This commonly-used scale is included in the appendix. For each question, the individual was asked to answer “Never” (0), “Sometimes” (1), “Most of the time” (2), “Almost always” (3), “Don’t know” (4). When scoring, all the “don’t know” were skipped, so each question was scored on a range of 0–3. The scores for each question were added together to create a total score of each individual. A total score of 0 is classified as non-problem gambling, a total score of between 1 and 2.5 is classified as low-risk gambling, a total score between 3 and 7.5 is classified as moderate-risk gambling and a total score of 8 or greater is classified as problem gambling. The scale had adequate internal consistency, with a Cronbach α’s of .96.

#### Psychological factors and erroneous gambling cognitions

In the survey, the psychological factors and erroneous gambling cognitions of depression (Cronbach α = .92 [67]), low esteem (Cronbach α = .88 [57]), positive urgency (Cronbach α = .91 [68, 69]), overestimating the chances of winning (OCW; Cronbach α = .88 [70]), luck/perseverance (Cronbach α = .94 [62]) were assessed. To avoid the survey becoming overly long, shorter versions of these scales that had been developed in a previous study [32] were used. Accordingly, each scale contained only four items.

## Pilot study

Before running the large-scale survey, the survey was piloted on 53 university students. This pilot confirmed that the online survey functioned as expected (i.e., it had no bugs), could be completed in an appropriate length of time, and was comprehensible. Further details regarding this pilot are available in the supplementary information.

## Results

Three thousand nine hundred and six Victorians were contacted by the ORU in June or July 2016. Of these, 3361 agreed to participate (86%). Participants ranged in age from 18 to 88 years (*M*_*age*_ = 46.7, *SD* = 16.7), 48% were male and 71% lived in the metropolitan area of Melbourne. The majority of participants reported that they were born in Australia (77%), were in a relationship (62%), and spoke English at home (94%). The median response time for this survey was 12.9 minutes.

To investigate which factors are associated with gambling participation rates, a correlation analysis was performed on each of the 12 gambling activities in turn (Table 1). It was found that all variables were significantly correlated with participation in at least some forms of gambling. Discussing gambling both online and offline was significantly correlated with gambling participation rates in all forms of gambling. This is noteworthy since no previous study has reported a correlation between discussing gambling and self-reported gambling participation rates.

**Table 1:** Spearman rank order coefficients (r_s_) between Victorians’ self-reported participation in 12 gambling activities and all variables of interest.

Variables	Self-reported participation in gambling activity											
	Lottery tickets	Instant scratch tickets	Raffle or fund-raising tickets	Betting on animal races	Sports betting	Gaming tables at casinos	Poker machines at casinos	Poker machines at other venues	Cards or board games	Games of skill	Arcade or video gaming	Internet gambling
	r_s_	r_s_	r_s_	r_s_	r_s_	r_s_	r_s_	r_s_	r_s_	r_s_	r_s_	r_s_
Participation in gambling												
Family members	.46**	.51**	.54**	.46**	.40**	.48**	.52**	.52**	.60**	.58**	.58**	.49**
Peers	.43**	.35**	.47**	.41**	.35**	.38**	.37**	.39**	.43**	.39**	.38**	.38**
People in general	.23**	.14**	.25**	.13**	.04*	.06**	.10**	.14**	.14**	.11**	.12**	.10**
Approval of gambling												
Self-reported	.49**	.34**	.35**	.49**	.48**	.33**	.38**	.49**	.29**	.24**	.26**	.46**
Family members	.36**	.29**	.30**	.33**	.31**	.22**	.27**	.32**	.25**	.21**	.20**	.29**
Peers	.26**	.20**	.28**	.25**	.24**	.18**	.18**	.23**	.23**	.17**	.17**	.26**
People in general	.15**	.06**	.21**	.03	.02	-.01	.04*	.11**	.13**	.05**	.09**	.09**
Exposure to advertisement												
Seeing ads	.24**	.19**	.35**	.25**	.08**	.21**	.20**	.16**	.30**	.35**	.42**	.18**
RPM	.18**	.25**	.31**	.33**	.38**	.37**	.33**	.28**	.37**	.47**	.50**	.31**
Participating in discussions												
Online	.10**	.29**	.19**	.33**	.41**	.42**	.34**	.27**	.47**	.54**	.57**	.42**
Offline	.36**	.42**	.38**	.48**	.43**	.44**	.41**	.42**	.55**	.58**	.60**	.41**
Demographic variables												
Age	.28**	-.06**	.15**	-.03	-.24**	-.22**	-.11**	.07**	-.22**	-.23**	-.24**	-.17**
Gender^a^	-.09**	.02	.03	-.16**	-.25**	-.14**	-.02	-.03	-.09**	-.12**	-.10**	-.17**
COB^a^	.00	.03	.07**	.10**	.04*	.01	-.01	.09**	-.05**	.00	.01	.04*
LSH^a^	.04*	.01	.09**	.07**	-.02	-.05**	-.03	.07**	-.07**	-.03	-.04**	-.01
Relationship status^a^	.14**	.07**	.15**	.06**	.03	.04*	.04*	.05**	.01	.01	.01	.00
Location^a^	-.01	-.02	-.08**	.02	.09**	.11**	.12**	-.02	.10**	.07**	.08**	.06**
Psychological factors												
Depression	-.06**	.09**	-.02	.07**	.15**	.16**	.13**	.12**	.18**	.23**	.24**	.21**
Low esteem	-.03	.09**	.00	.07**	.16**	.17**	.13**	.12**	.19**	.25**	.26**	.22**
Positive urgency	.02	.18**	.05**	.18**	.28**	.30**	.24**	.20**	.31**	.35**	.35**	.30**
OCW	.14**	.25**	.06**	.23**	.29**	.31**	.29**	.24**	.26**	.29**	.29**	.26**
Luck/Perseverance	.04*	.16**	.07**	.11**	.18**	.19**	.16**	.13**	.17**	.22**	.21**	.17**
PGSI score	.20**	.29**	.13**	.38**	.46**	.45**	.41**	.45**	.34**	.37**	.36**	.45**

*Note*. *N* = 3361. COB = Country of birth; LSH: Main language spoken at home; OCW = Overestimating chances of winning, PGSI = Problem gambling severity index, PRM = Receiving promotional material.

COB^a^: 0 = Other, 1 = Australia.

Gender^a^: 0 = male, 1 = female.

Location^a^: 0 = Rural, 1 = Metropolitan.

LSH^a^: 0 = Other, 1 = English.

Relationship status^a^: 0 = Other, 1 = Married or living with a partner.

*p < .05.

**p < .01.

***p < .001.

A linear regression was performed to estimate to how much of the variance of gambling participation rates each factor uniquely predicts. As shown in Table 2, for all gambling activities, the regression fit was highly significant with *p* < .001 and with each regression explaining between 41% and 51% of the variance in the data.

**Table 2:** Model summary statistics for a series of linear regression analyses predicting Victorians’ self-reported participation rates in each of 12 gambling activities.

Gambling activity	Self-reported participation in gambling activity			
	R^2^	F	df_Reg_, df_Res_	*p*
Lottery tickets	.48	131.45	23, 3324	< .001
Instant scratch tickets	.41	99.83	23, 3324	< .001
Raffle or fund-raising tickets	.42	105.37	23, 3324	< .001
Betting on animal races	.47	129.96	23, 3324	< .001
Sports betting	.47	127.85	23, 3324	< .001
Gaming tables at casinos	.45	116.06	23, 3324	< .001
Poker machines at casinos	.42	106.54	23, 3324	< .001
Poker machines at other venues	.49	141.43	23, 3324	< .001
Cards or board games	.51	148.14	23, 3324	< .001
Games of skill	.50	145.95	23, 3324	< .001
Arcade or video gaming	.50	143.59	23, 3324	< .001
Internet gambling	.45	116.07	23, 3324	< .001

*Note*. *N* = 3361. Predictors: (i) Beliefs about the frequency of participation of family members, peers, and people in general; (ii) Self-reported approval of gambling and beliefs about the approval of gambling of family members, peers, and people in general; (iii) Exposure to gambling advertisement; (iv) Participation in discussions about gambling; (v) Demographics variables (age, gender, country of birth, language spoken at home, location, relationship status), and (vi) Psychological factors (depression, low esteem, positive urgency, overestimating chances of winning, luck/ perseverance, problem gambling severity index score).

Table 3 shows that most factors that are known to be correlated with gambling frequency were, at most, minor regression predictors of gambling frequency. The most significant regression predictors were the degree to which family members and peers were perceived to gamble, self-reported approval of gambling, participation in offline discussions of gambling, and PGSI score. In addition, age was an important predictor of gambling for lottery tickets and, to a lesser extent, raffle tickets and gambling on poker machines.

**Table 3:** Standardised regression coefficients for the predictors of self-reported participation in 12 gambling activities.

Predictors	Self-reported participation in gambling activity											
	Lottery tickets	Instant scratch tickets	Raffle or fund-raising tickets	Betting on animal races	Sports betting	Gaming tables at casinos	Poker machines at casinos	Poker machines at other venues	Cards or board games	Games of skill	Arcade or video gaming	Internet gambling
	β	β	β	β	β	β	β	β	β	β	β	β
Participation in gambling												
Family members	.23***	.30***	.30***	.18***	.13***	.22***	.28***	.26***	.38***	.29***	.31***	.23***
Peers	.17***	.10***	.16***	.12***	.11***	.14***	.13***	.12***	.10***	.07***	.07***	.11***
People in general	.02	.03	.01	.00	-.01	.00	.02	.04**	-.01	-.01	.00	.04*
Approval of gambling												
Self-reported	.37***	.20***	.22***	.30***	.32***	.16***	.23***	.34***	.11***	.05*	.07***	.35***
Family members	-.02	.02	.00	-.02	-.03	-.03	-.03	-.07***	-.02	.01	-.03	-.07***
Peers	-.09***	-.03	-.05*	-.04*	-.08***	-.06**	-.07***	-.06***	-.02	-.03	-.02	-.02
People in general	-.10***	-.08***	-.05*	-.08***	-.05**	-.06***	-.05**	-.03	-.01	-.01	-.03	-.06***
Exposure to advertisement												
Seeing ads	.01	.00	.08***	.04**	-.02	.01	.00	-.03*	.01	.02	.06***	.03*
RPM	.03	.03	.06***	.07***	.11***	.07***	.06***	.05**	.03	.06***	.03*	.03*
Participating in discussions												
Online	-.01	.04*	.02	.03	.05**	.08***	.03	-.04*	.02	.03	.05**	.01
Offline	.16***	.19***	.12***	.23***	.18***	.14***	.13***	.17***	.23***	.25***	.24***	.13***
Demographic variables												
Age	.21***	.02	.11***	.03*	-.03*	-.04**	.01	.12***	-.02	-.01	-.03*	.02
Gender^a^	-.07***	-.01	.00	-.08***	-.14***	-.06***	.01	.00	-.03**	-.05***	-.03*	-.06***
COB^a^	.01	.00	.02	.02	.02	.02	-.01	.03*	-.02	.00	.00	.01
LSH^a^	-.03*	-.01	.00	.00	-.02	-.03	-.01	.00	.00	.00	.00	.00
Relationship status^a^	.05***	.04**	.07***	.02	.01	.03*	.01	.01	.02	.02	.02	-.01
Location^a^	.00	-.01	-.04**	.02	.03*	.03*	.05***	-.02	.02	.01	.02	.02
Psychological factors												
Depression	-.06*	.01	-.05**	.02	-.04	.00	.02	.01	-.01	.01	.01	.01
Low esteem	.01	-.05	.01	-.06*	-.01	-.06*	-.06*	-.05	-.04	-.02	-.01	-.02
Positive urgency	-.01	.00	.05*	-.03	-.01	.03	-.02	.02	.04*	.04*	.04*	.00
OCW	.10***	.10***	.07***	.07***	.05**	.07***	.08***	.05**	.04*	.03	.01	.01
Luck/ Perseverance	-.05***	.00	.00	-.04**	-.02	.01	-.01	-.05***	.01	.04**	.01	-.02
PGSI	.10***	.11***	.03	.13***	.17***	.18***	.19***	.24***	.10***	.15***	.08***	.19***

*Note*. *N* = 3361. COB = Country of birth; LSH: Main language spoken at home; OCW = Overestimating chances of winning, PGSI = Problem gambling severity index, PRM = Receiving promotional material.

COB^a^: 0 = Other, 1 = Australia.

Gender^a^: 0 = male, 1 = female.

Location^a^: 0 = Rural, 1 = Metropolitan.

LSH^a^: 0 = Other, 1 = English.

Relationship status^a^: 0 = Other, 1 = Married or living with a partner.

*p < .05;

**p < .01;

***p < .001

**Fig 1:**
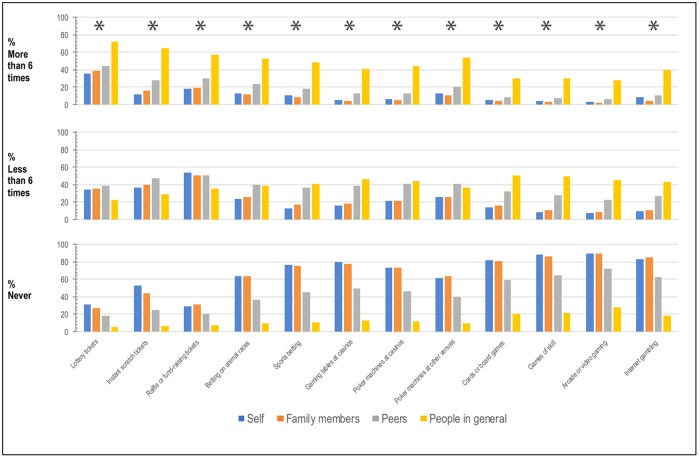
Clustered bar graphs for the percentage of Victorians self-reporting participating in the 12 surveyed gambling activities and the perceived participation of their family members, peers, and people in general. Clusters marked with asterisks indicate a statistically significant difference on frequency ratings for gambling activity depending on the person(s) doing the gambling (self, family members, peers, and people in general) as indicated by a non-parametric Friedman test. *N* = 3361. *p < .001.

**Fig 2:**
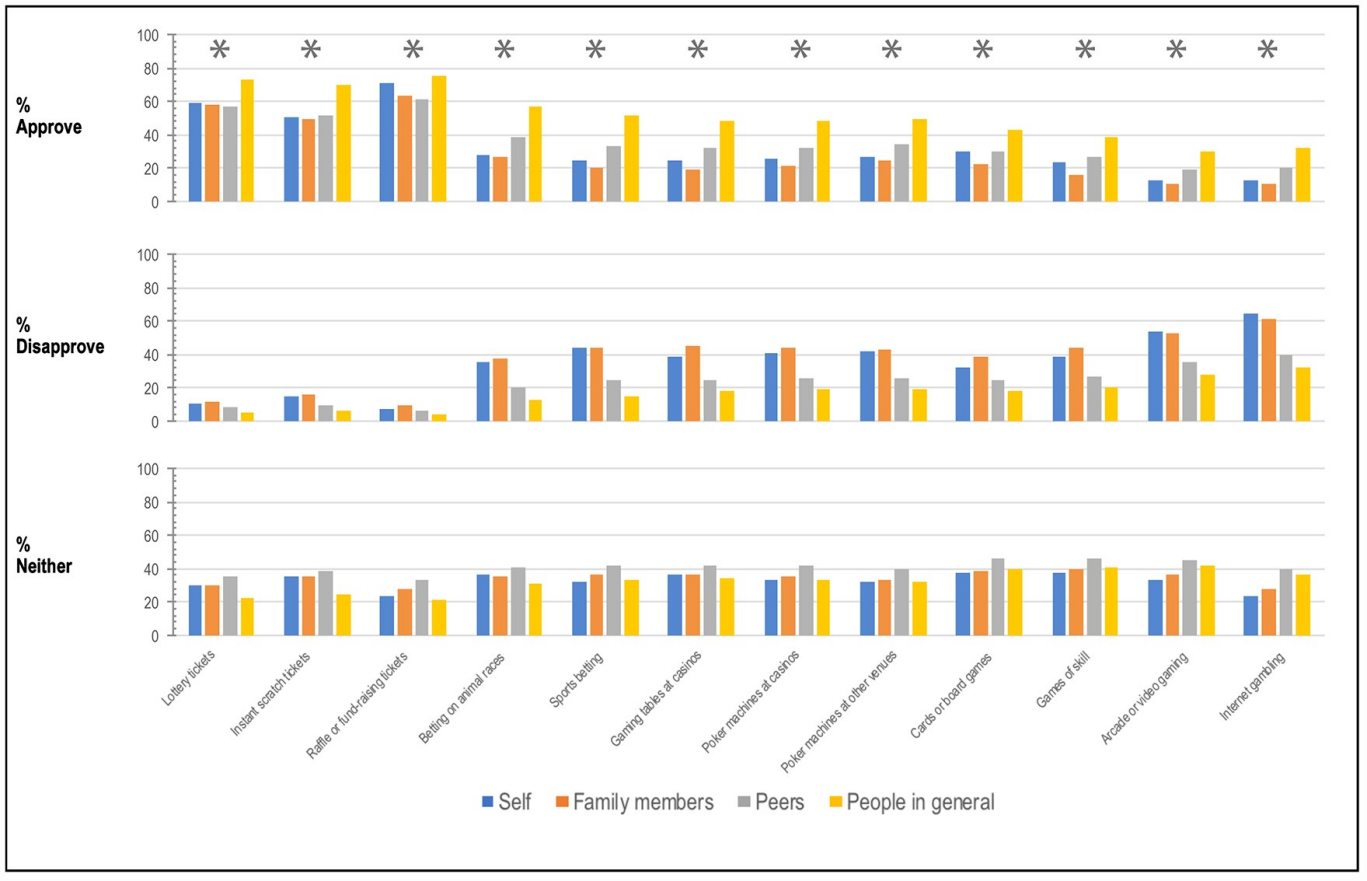
Clustered bar graphs showing the percentage of Victorians who approve of each of the 12 gambling activities and what percentage of their family members, peers, and people in general, respectively, they believe approve of each of the 12 gambling activities. Clusters marked with asterisks indicate a statistically significant difference in approval ratings as a function of approval category (self, family members, peers, and people in general), as indicated by a non-parametric Friedman test. *N* = 3361. *p < .001.

Since these data show that beliefs about the degree to which others gamble and approval of gambling are important regression predictors of gambling frequency, it is worthwhile considering how accurate these beliefs were. Fig 1 shows the average self-reported gambling frequency versus the perceived gambling frequency of family, peers, and people in general, for each of the 12 gambling activities. There is an incongruence between these two estimates, with the perceived gambling frequency of others being systematically greater than the average self-reported gambling frequency. Fig 2 shows the degree to which individuals perceived family members, peers, and people in general to approve of gambling. There is a systematic bias for individuals to believe that people in general approve of gambling more than people self-report that they do.

Table 4 shows that almost all variables were correlated with PGSI score, consistent with the previous literature. The only exceptions were the variables, *Language spoken at home* and *Relationship status*.

**Table 4:** Spearman rank order coefficients (r_s_) between PGSI score and all variables of interest.

Variables	PGSI score											
	Lottery tickets	Instant scratch tickets	Raffle or fund-raising tickets	Betting on animal races	Sports betting	Gaming tables at casinos	Poker machines at casinos	Poker machines at other venues	Cards or board games	Games of skill	Arcade or video gaming	Internet gambling
	r_s_	r_s_	r_s_	r_s_	r_s_	r_s_	r_s_	r_s_	r_s_	r_s_	r_s_	r_s_
Participation in gambling												
Self-reported	.20**	.29**	.13**	.38**	.46**	.45**	.41**	.45**	.34**	.37**	.36**	.45**
Family members	.10**	.16**	.07**	.20**	.25**	.26**	.24**	.24**	.26**	.29**	.32**	.30**
Peers	.07**	.09**	.04*	.17**	.21**	.23**	.21**	.20**	.18**	.19**	.18**	.22**
People in general	-.11**	-.09**	-.08**	-.05**	-.03*	-.02	-.04*	-.05**	-.02	-.02	-.02	-.03
Approval of gambling												
Self-reported	.05**	.05**	-.05**	.24**	.26**	.27**	.27**	.27**	.15**	.20**	.20**	.31**
Family members	-.02	-.01	-.09**	.10**	.14**	.12**	.10**	.12**	.08**	.11**	.11**	.15**
Peers	-.03	-.01	-.05**	.08**	.12**	.12**	.10**	.10**	.07**	.09**	.09**	.13**
People in general	-.12**	-.13**	-.13**	-.08**	-.06**	-.05**	-.07**	-.08**	-.05**	-.06**	-.05**	-.01
Exposure to advertisement												
Seeing ads	.01	.09**	.05**	.06**	.05**	.16**	.16**	.15**	.23**	.22**	.23**	.10**
RPM	.25**	.25**	.15**	.30**	.31**	.33**	.33**	.29**	.29**	.30**	.30**	.28**
Participating in discussions												
Online	.36**	.35**	.30**	.36**	.37**	.38**	.37**	.36**	.34**	.35**	.34**	.36**
Offline	.24**	.25**	.18**	.30**	.29**	.34**	.33**	.32**	.33**	.33**	.35**	.30**
	Demographic variables							Psychological factors				
	Age	Gender^a^	COB^a^	LSH^a^	Relationship status^a^	Location^a^		Depression	Low esteem	Positive urgency	OCW	Luck/ Perseverance
PGSI score	-.18**	-.18**	.05**	-.01	-.03	.06**		.33**	.35**	.46**	.43**	.25**

*Note*. *N* = 3361. COB = Country of birth; LSH: Main language spoken at home; OCW = Overestimating chances of winning, PGSI = Problem gambling severity index, PRM = Receiving promotional material.

COB^a^: 0 = Other, 1 = Australia.

Gender^a^: 0 = male, 1 = female.

Location^a^: 0 = Rural, 1 = Metropolitan.

LSH^a^: 0 = Other, 1 = English.

Relationship status^a^: 0 = Other, 1 = Married or living with a partner.

*p < .05;

**p < .01;

***p < .001.

Whereas in the previous regression analysis only one form of gambling was considered at a time, this would not be an appropriate way of predicting PGSI score, since multiple forms of gambling could potentially contribute to an individual’s PGSI score. To account for multiple forms of gambling simultaneously, a stepwise regression was performed. First the single predictor of PGSI score that could explain the greatest amount of variance in PGSI score was identified. Then, the second predictor which, when combined with the first predictor, explained the greatest amount of variance in PGSI score was found. In the next step, a third predictor was added and so on, so that each step added another predictor. This process continued until adding an additional predictor did not explain a statistically significant greater amount of variance. Doing this, revealed 33 regression predictors which, in total, explained 56% of the variance in PGSI score. However, almost all this variance was accounted for by the top ten regression predictors which, combined, explained 54% of the variance of the PGSI score. Consequently, Table 5 shows just these regression predictors.

**Table 5:** Summary statistics for a stepwise linear regression designed to predict PGSI score.

Gambling activity	PGSI score			
	R^2^	F	df_Reg_, df_Res_	*p*
1. Psychological factor: Positive urgency	.343	1743.58	1, 3346	< .001
2. Self-reported participation: Playing poker machines at pubs, hotels, or sporting clubs	.421	1214.19	1, 3345	< .001
3. Participation in online discussion about: Betting on gaming tables at casinos	.464	965.43	1, 3344	< .001
4. Self-reported participation: Gambling on the Internet	.492	809.82	1, 3343	< .001
5. Psychological factor: Overestimating chances of winning	.512	702.56	1, 3342	< .001
6. Psychological factor: Low esteem	.519	601.97	1, 3341	< .001
7. Perceived participation of family members: Betting on arcade or video games	.525	528.26	1, 3340	< .001
8. Perceived participation of people in general: Buying lottery tickets such as Tattslotto, Powerball, or Keno	.529	467.87	1, 3339	< .001
9. Participation in online discussion about: Playing poker machines at casinos	.532	421.27	1, 3338	< .001
10. Perceived participation of family members: Playing poker machines at pubs, hotels, or sporting clubs	.535	383.81	1, 3337	< .001

## Discussion

The aim of this study was to determine which factors are the most important predictors of gambling frequency and problem gambling. It was found that the major predictors of gambling frequency were the degree to which family members and peers were perceived to gamble, self-reported approval of gambling, participation in offline discussions of gambling, and PGSI score. In addition, age was an important predictor of gambling frequency for some forms of gambling (e.g., lottery tickets). Because the degree to which others are perceived to gamble was one of the strongest regression predictors of gambling frequency, the study also investigated the accuracy of the perceptions of the degree to which others gamble and approve of gambling. Consistent with Larimer and Neighbors [12], it was found that, relative to self-reports, individuals overestimated how much others gamble and overestimated how much they approved of gambling. This suggests that campaigns aimed at reducing gambling would do well to focus on correcting these discrepancies. Additionally, such campaigns should use personalised norms, as such norms appear to be particularly effective at affecting gambling behaviour [71].

The study also investigated the factors correlated with PGSI score. Although most of the factors that were correlated with gambling frequency were also correlated with PGSI score, *Language spoken at home* and *Relationship status* were not correlated with PGSI score. This is possibly due, at least partly, to the fact that both factors were only weakly correlated with gambling frequency. It is also possible that the reason why *Language spoken at home* was not correlated with PGSI score was that the panel members for the survey company would likely have very good English language skills, which would reduce the effect of speaking a different language at home. It is also possible that the reason why *Relationship status* was not correlated with PGSI score was that gambling is so normalised in the Australian culture that spouses are tolerant of gambling problems.

It was found that 33 variables were significant regression predictors and, in total, could explain 56% of the variance in PGSI score. However, the majority (91%) of the explainable variance could be explained by just the first five regression predictors. These were: *Positive urgency; Playing poker machines at pubs*, *hotels*, *or sports clubs; Online discussions of gaming tables at casinos; Gambling on the internet*; and *Overestimating chances of winning*. This suggests that interventions designed to reduce problem gambling should concentrate on these factors. In the state of Victoria there is already a movement to reduce access to poker machines in pubs, hotels and sports clubs. It would be beneficial if access to poker machines in these venues could be reduced further. Furthermore, interventions that reduce the degree to which people overestimate the chances of winning would also help. For example, educating people to avoid common gamblers fallacies might help reduce problem gambling. Finally, when treating problem gamblers, these results suggest that counsellors should consider concentrating on reducing and better controlling positive urgency.

The prevalence of problem gambling found in the current study (11%) is considerably higher than that found in other surveys of the Victorian population (0.8% [4]; 0.6% [5]). It is possible that the elevated prevalence rate in the current survey may, to some extent, be explained by the fact that the participants understood that their responses would be completely anonymous, as opposed to merely being confidential, which would be expected to elicit more honest answers [65]. The rest of difference may be due to the survey not being completely representative, as it was necessarily limited to those people with access to the internet [72]. Future work will need to determine the cause of this discrepancy so as to establish a more reliable estimate of the prevalence of problem gambling in Victoria.

### Limitations

Because our survey was conducted online, it cannot be claimed to be representative of the general Victorian population, as it was necessarily limited to those people with access to the internet [72]. It was also administered by a survey company, the ORU, to a panel of individuals who had previously agreed with this survey company to answer online surveys. The above not withstanding, we took steps to make our survey as representative as possible. Specifically, the recruitment email sent by the ORU did not specify the nature of our survey (i.e., it did not state that it was a gambling survey), so as to avoid biasing our recruitment towards over-representing gamblers and those interested in gambling. In addition, our sample was matched to that of the general Victorian population in terms of gender, age and location, as detailed in the supplementary materials. However, the survey itself was clearly focused on gambling, so it is possible that this may have biased the responses of our participants.

Additionally, the fact that some of the predictors are correlated, reduces the stability of the results. Although the fact that a large number of participants were surveyed to some extent mitigates this issue [73], future work is needed to determine to what extent the results will generalize, especially to different populations. It should also be acknowledged that, to increase the chance that contacted individuals would agree to participate, a relatively short survey was used which did not include some factors that could have been associated with gambling frequencies and gambling problems. Consequently, it could be that a factor that the analysis indicates is an important predictor of either gambling frequency or PGSI score would no longer be found to be an important predictor when these other factors are included. A final limitation is that the frequency data included only two time periods (i.e., more than six times and less than six times in the past year) in addition to never. It is possible that using more categories for the frequencies of behaviour, for seeing advertisements and for receiving promotional materials could have allowed a more nuanced description of the extent to which other people are perceived to gamble and the impact of advertisements and promotional materials on gambling behaviour and gambling problems.

## Conclusions

Although previous research has shown that a large number of factors are correlated with gambling frequency, it was unclear from that research to what extent those factors could predict gambling frequency or problem gambling. As such, it was unclear what the primary drivers of gambling are, so it was unclear on which factors future research should focus. The main finding of the current study was that only some of the factors that are correlated with gambling frequency actually predict either gambling frequency or problem gambling, beyond that which can be predicted by other factors. Future work should focus on these major predictors as they are likely to be the most important factors driving gambling frequency and problem gambling.

There are a number of practical implications of these findings for gambling in Australia. First, since the frequency of playing poker machines is a major predictor of problem gambling, interventions that reduce access to poker machines are likely to reduce problem gambling rates. In the state of Victoria there is already a movement to do this, which should help reduce problem gambling. Additionally, it would be useful if the state were to implement an information campaign to reduce the degree to which people overestimate the chances of winning. Such a campaign could aim to educate people to avoid common gambling fallacies. Finally, these findings suggest that when treating problem gamblers, counsellors should concentrate on reducing and better controlling positive urgency.

## Supporting information

S1 Appendix Description of the pilot study. (DOCX)

S2 Appendix Survey demographics. (DOCX)

S3 Appendix Questionnaire. (DOCX)

S4 Appendix Anonymized data. (SAV)
