# Supplementary material for: Predictors of gambling and problem gambling in Victoria, Australia
Source: PLoS One. 2019 Jan 23;14(1):e0209277. doi: 10.1371/journal.pone.0209277 (PMC6343914; doi:10.1371/journal.pone.0209277)
Supplement: S1 Appendix — (DOCX) [file pone.0209277.s001.docx]

## Pilot Study

Our research strategy was to first carry out a pilot study, using a small sample of university students. The purpose of this pilot study was to determine if there were any problems or misunderstandings with any of our survey items and if our survey was an appropriate length. Our survey company, the Online Research Unit (ORU), had informed us that if our survey were to take longer than 20 minutes, it would likely have an unacceptably high drop-out rate. It was therefore important that our survey took the majority of participants less than 20 minutes to complete while simultaneously ensuring that all its items were readily comprehensible.

## *Method*

### *Participants*

Fifty-three undergraduate students were recruited, but one dropped out before completing the survey, so is not included in our analysis. Of the remaining participants, 21 were males (40%) and all were aged between 18 and 29 years (mean age 19.52 years, *SD* = 2.53). Participants were paid $12 to compensate them for their time.

### *Procedure*

The participants were presented with our survey using the Qualtrics online survey platform. The survey questions are listed below. Once the participants had completed the survey, they then discussed the questions with the research assistant. These discussions were designed to uncover any difficulties or potential misunderstandings with the survey items. The survey data itself was not analysed

## *Results*

Of the 52 students who completed our pilot study, only three (6%) indicated any difficulties with the survey questions. These students commented:

“I find that it is sometimes hard to provide an accurate response for what I perceive the general people think of gambling and it is sometimes difficult for me to recall activities that I have engaged with 6 to 12 months ago.”

“The questions about how often I thought the general public gambled etc. were a bit difficult as I found it hard to consider what was meant by the general public, and I suppose I don't know that much about gambling habits of Australia overall. The question that asked me to answer questions as a gambler: I was unsure if it meant to answer them as what I perceived of most gamblers (I answered like this), or that I should answer them as if how I would view them if I personally gambled.”

“I found the wording of the questions about responding to statements as though you were a gambler a little confusing.”

Accordingly, given that 94% of the student sample had no difficulties with questions, no items were changed for the main study. Further, almost all (92%) individuals completed the survey in under 20 minutes, with a median time of 11.4 minutes.

## *Conclusions*

The results of this pilot study indicated that our survey questions were comprehensible and were appropriate for use in our larger field study. Further, most participants completed it in under 20 minutes, so it satisfied the requirements of our recruitment company, the ORU.
