# Supplementary material for: Predictors of gambling and problem gambling in Victoria, Australia
Source: PLoS One. 2019 Jan 23;14(1):e0209277. doi: 10.1371/journal.pone.0209277 (PMC6343914; doi:10.1371/journal.pone.0209277)
Supplement: S2 Appendix — (DOCX) [file pone.0209277.s002.docx]

## Survey Demographics

In recruiting participants, the ORU attempted to match our sample to the Victorian population in general for age, gender and location (Metropolitan versus regional), according to the demographic data obtained from the 2011 Australian Bureau of Statistics (ABS) survey. The data from the 2016 ABS survey was not used as it had not been released when the present study was run. It was not possible to perfectly match on any single dimension, given the time constraints, the finite size of the subject panel and the fact that the ORU was trying to match simultaneously on three different dimensions (gender, age and location).

**S1 Table. Gender.**

|  | **ABS 2011** | **Our survey** |
| --- | --- | --- |
| **Male** | 49.4% | 48.1% |
| **Female** | 50.6% | 51.8% |

**S2 Table. Location.**

|  | **ABS 2011** | **Our survey** |
| --- | --- | --- |
| **Melbourne** | 75.4% | 70.7% |
| **Regional** | 24.6% | 29.3% |

**S3 Table. Age.**

|  | **ABS 2011** | **Our survey** |
| --- | --- | --- |
| **18-24** | 12.43% | 11.54% |
| **25-29** | 8.46% | 8.63% |
| **30-34** | 9.28% | 9.02% |
| **35-39** | 9.71% | 8.39% |
| **40-44** | 9.75% | 8.42% |
| **45-49** | 9.59% | 8.75% |
| **50-54** | 8.72% | 8.12% |
| **55-59** | 8.18% | 8.75% |
| **60-64** | 6.35% | 10.38% |
| **65+** | 17.53% | 18.00% |
