# Supplementary material for: Predictors of gambling and problem gambling in Victoria, Australia
Source: PLoS One. 2019 Jan 23;14(1):e0209277. doi: 10.1371/journal.pone.0209277 (PMC6343914; doi:10.1371/journal.pone.0209277)
Supplement: S3 Appendix — (DOCX) [file pone.0209277.s003.docx]

## Questionnaire

The same questionnaire was used in both the pilot study and in the large-scale survey of Victorian adults. It was presented to participants online via the Qualtrics platform. The questionnaire was as follows.

[First page]

There are location requirements that must be met in order to participate in our study. Please type your postcode on the box below, then click "next".

If you do not meet the criteria the survey will terminate and you will be redirected to the screen out page.

PostCode: [Free response]

[New page – assuming participants meet location requirements]

**Melbourne School of Psychological Sciences

Plain Language Statement**

PROJECT TITLE:  **The Evolution and Consequences of the Perceived Normalisation of Gambling in Victoria**

**Dr. Piers Howe (Principal Researcher)** Tel: +61 3 8344 6287; email: [pdhowe@unimelb.edu.au](mailto:pdhowe@unimelb.edu.au)
**A/Prof Jennifer Boldero (co-PI)**email: [bolderoj@unimelb.edu.au](mailto:bolderoj@unimelb.edu.au)
**A/Prof Carol Hulbert (co-PI)** email: [cah@unimelb.edu.au](mailto:cah@unimelb.edu.au)
**Ms Adriana Vargas (co-PI)**email: [adriana.vargas@unimelb.edu.au](mailto:adriana.vargas@unimelb.edu.au)

**Introduction**

We would like to invite you to participate in a project that investigates the evolution and consequences of the perceived normalisation of gambling in Victoria. This research is funded in part by the Victorian Responsible Gambling Foundation.

**What I will be asked to do?**

If you decide to participate, you would answer a series of questions on a survey. These questions cover topics such as your age and gender, your gambling habits, your perception of the degree to which gambling has been normalised in society, your perception of the degree to which you perceive yourself to be lucky, your perceived chances at winning at gambling, your mood and self-esteem, the degree to which your mood affects your actions, the frequency with which you are exposed to gambling advertisements and promotions, and the frequency that you discuss gambling and partake in simulated gambling (i.e. gambling without wagering money).

**What are the risks?**

It is very unlikely that answering this survey will have a negative impact on your mental health. However, if on reflecting on your gambling habits you decide that you may have a gambling problem we would encourage you to contact the Victorian Responsible Gambling Foundation for support ([www.responsiblegambling.vic.gov.au](http://www.responsiblegambling.vic.gov.au/)). Similarly, if on reflecting on your mood you decide that you may be depressed we would encourage you to contact the University of Melbourne Psychology Clinic ([www.psych.unimelb.edu.au/about/psychology-clinic](http://www.psych.unimelb.edu.au/about/psychology-clinic)) or Lifeline ([www.lifeline.org.au](http://www.lifeline.org.au/)). Alternatively, you should feel free to contact Dr. Piers Howe using the contact details listed above.

**How will my confidentiality be protected?**

Any data that you provide will be kept in password-protected computer files, accessible only by the named researchers. You will not be referred to by name in any publications arising from this study and all data will be kept strictly confidential, subject to any legal limitations. The data will be maintained for a minimum of five years after publication.

**What if I want to withdraw from the research?**

Participation in this research is completely voluntary. You are free to withdraw at any time and to withdraw any unprocessed data previously supplied. This would have no effect on your relationship with any member of the School of Psychological Sciences at the University of Melbourne and will not affect any ongoing assessment, grades, or treatment.

**Where can I get further information?**

If you have not understood any of this information please contact any of the researchers listed above. This research has been cleared by the Human Research Ethics Committee (HREC 1545085). If you have any concerns about this project please contact the Executive Officer, Human Research Ethics, The University of Melbourne (Tel: +61 3 8344 2073; Fax: +61 3 9347 6739).

**How do I agree to participate?**

If you wish to participate please read and indicate you agreement with the consent form that you will now be shown.

[New page]

**Melbourne School of Psychological Sciences

Consent form for persons participating in a research project**

PROJECT TITLE:  **The Evolution and Consequences of the Perceived Normalisation of Gambling in Victoria**

Name of Primary Researcher: Dr. Piers Howe
Name of Additional Researchers: A/Prof Jennifer Boldero (co-PI), A/Prof Carol Hulbert (co-PI), Ms. Adriana Vargas (co-PI),

1. I consent to participate in this project. The purpose of this research is to investigate the evolution and consequences of the perceived normalisation of gambling in Victoria.
 
2. I understand that this project is for research purposes only and not for treatment.
 
3. I understand that this research is funded in part by the Victorian Responsible Gambling Foundation.
 
4. In this project I will be asked to respond to a survey. The details of this have been explained in the Plain Language Statement, of which I have been given the opportunity to make a copy.
 
5. I understand that it is highly unlikely that responding to this survey will have any adverse affects on my mental health. However, by reflecting on my mood and my gambling habits it is possible that I may decide that I may be depressed and/or have a gambling problem. The Plain Language Statement has listed a number of resources that I can access if this occurs. Alternatively, I am free to contact the primary researcher, Dr. Howe.
 
6. My participation is voluntary and that I am free to withdraw from the project at any time without explanation or prejudice and to withdraw any unprocessed data I have provided. Withdrawing from the project will not affect my relationship with the School of Psychological Sciences at the University of Melbourne in any way and will not affect any ongoing assessment, grades, or treatment.
 
7. I have been informed that the data from this research will be stored at the University of Melbourne and will be kept for at least five years from the date of publication of the results of this study.
 
8. I have been informed that the confidentiality of the information I provide will be safeguarded subject to any legal requirements; my data will be password protected and accessible only by the named researchers.
 
9. I understand that after I indicate consent, a record of my consent will be retained by the researcher.

If you wish to consent to this study, please tick the **"Continue"** box below.  If you wish to withdraw consent to take part in this study, please tick the **"Discontinue"** box below. Note that you if you decide to click the "Discontinue" box, you will be exiting the study at this stage.

Once you have made your choice, click the "Next" button to continue.

Continue

Discontinue

[New page]

Section 1- **Demographics**

In this section we would like to find out about you and your household. We are interested in collecting this information so we can make comparisons between different groups of individuals, different households, and different geographical areas.

What is the street name, suburb, and postcode of your property? (Please DO NOT include your street number or lot number)

Street: [Free response]

Suburb: [Free response]

Postcode: [Free response]

What gender do you identify with? [Choice of “Male”, “Female”, “Other (please specify)]

Please specify your age in years: [Free response]

Where were you born? [Choice of “Australia”, “Other (please specify)”]

What is the main language you speak at home? [Choice of “English”, “Other (please specify)”]

What is your marital status? [Choice of “Married”, “Living with a partner (e.g. a de facto, or boyfriend/girlfriend)”, “Single, that is, never married”, “Separated (still legally married)”, “Divorced”, “Widowed”]

Section 2 - **Gambling Behaviour (PGSI)**

We would now like to ask you some questions **about YOUR gambling behaviour. Thinking about the last 12 months,** please answer the following questions.

[For the following questions the choices were “Never” (0), “Sometimes” (1), “Most of the time” (2), “Almost always” (3), “Don’t know” (4). When scoring, all “don’t know” were skipped]

Have you bet more than you could really afford to lose?

Have you needed to gamble with larger amounts of money to get the same feeling of excitement?

When you gambled, did you go back another day to try to win back the money you lost?

Have you borrowed money or sold anything to get money to gamble?

Have you felt that you might have a problem with gambling?

Has gambling caused you any health problems, including stress or anxiety?

This is a catch question to check that you are reading the survey. Please click “Most of the time” to show that you have read this question.

Have people criticized your betting or told you that you had a gambling problem, regardless of whether or not you thought it was true?

Has your gambling caused any financial problems for you or your household?

Have you felt guilty about the way you gamble or what happens when you gamble?

Have you lied to family members or others to hide your gambling?

Have you bet or spent more money that you wanted to on gambling?

Have you wanted to stop betting money or gambling, but did not think you could?

Section 3 - **Approval of Gambling**

Please indicate **the extent YOU approve** of the following gambling activities.

[For the following questions the choices were “Strongly disapprove”, “Moderately disapprove”, “Neither approve nor disapprove”, “Moderately approve”, “Strongly approve”]

Buying lottery tickets such as Tattslotto, Powerball, or Keno?

Buying instant scratch tickets (a 'scratchie')?

Buying raffle or fundraising tickets?

Betting on horse races, trots or dog races?

Betting on sports like football, tennis, rugby or cricket?

Betting on gaming tables at casinos?

Playing poker machines at casinos?

Playing poker machines at pubs, hotels or sporting clubs?

Betting on cards or boards games with family or friends?

Betting on games of skill such as pool, bowling or darts?

Betting on arcade or video games?

Gambling on the Internet?

Section 3 - **Approval of Gambling**

Please indicate **the extent you think that YOUR FAMILY approves** of the following gambling activities.

Same list of questions and same scale as above.

Section 3 - **Approval of Gambling**

Please indicate **the extent you think that YOUR PEERS approve** of the following gambling activities.

Same list of questions and same scale as above.

Section 3 - **Approval of Gambling**

Please indicate **the extent you think that PEOPLE IN GENERAL approve** of the following gambling activities.

Same list of questions and same scale as above.

Section 4 - **Participation in Gambling**

Please indicate **the extent to which YOU have done** the following **within the last 12 months**.

[For the following questions the choices were “More than 6 times”, “Less than 6 times”, “Never”]

Bought a lottery ticket such as Tattslotto, Powerball, or Keno?

Bought an instant scratch ticket (a 'scratchie')?

Bought raffle or fundraising tickets?

Bet on horse races, trots or dog races?

Bet on sports like football, tennis, rugby or cricket?

Bet on gaming tables at a casino?

Played poker machines at a casino?

Played poker machines at a pub, hotel or sporting club?

Bet on cards or board games with family or friends?

Bet on games of skill such a pool, bowling or darts?

Bet on arcade or video games?

Gambled on the Internet?

Section 4 - **Participation in Gambling**

Please indicate **the extent you think YOUR FAMILY has done** the following **within the last 12 months.**

Same list of questions and same scale as above.

Section 4 - **Participation in Gambling**

Please indicate **the extent you think YOUR PEERS have done** the following **within the last 12 months.**

Same list of questions and same scale as above.

Section 4 - **Participation in Gambling**

Please indicate **the extent you think PEOPLE IN GENERAL have done** the following **within the last 12 months.**

Same list of questions and same scale as above.

Section 5 - **Views About Gambling**

Whether they gamble or not, people have views about gambling. Please rate **the extent to which YOU agree with** the following statements.

[For the following questions the choices were “Strongly disapprove”, “Moderately disapprove”, “Neither approve nor disapprove”, “Moderately approve”, “Strongly approve”]

Sometimes I just know I'm going to have good luck.

If I had lost my bets recently, my luck would be bound to change.

Sometimes I think I have the power to "will" my numbers to come up in gambling games.

If I concentrate hard enough I would be able to influence whether I win if I play poker machines.

This is a catch question to check that you are diligently reading the survey. Please tick “Neither agree nor disagree” to show you have read this question.

Section 5 - **Views About Gambling**

Regardless of whether you personally gamble, please **rate the extent to which YOU agree or disagree with** the following statements **as if you were a gambler.**

[For the following questions the choices were “Strongly disapprove”, “Moderately disapprove”, “Neither approve nor disapprove”, “Moderately approve”, “Strongly approve”]

Where I get money to gamble doesn't matter because I will win and pay it back.

If I continue to gamble, it will eventually pay off and I will make money.

I should keep the same bet even when it has not come up lately because it is bound to win.

There are certain things that I can do when I am betting - for example, tapping a certain number of times, holding a lucky coin in my hand, crossing my fingers, etc. - which increase the chances that I win.

Section 6 - **Perceptions of One's Self**

People see their lives in different ways. Please rate **the extent to which YOU agree that** each of the following statements **describe you.**

[For the following questions the choices were “Rarely or none of the time”, “A little”, “Sometimes”, “Often”, “Always or most of the time”]

I feel lonely.

When I am in a great mood I tend to get into situations that could cause me problems.

I am miserable to be around.

When I am very happy, I can't seem to stop myself from doing things that can have bad consequences.

I feel depressed.

When overjoyed, I feel as like I can't stop myself from going overboard.

I am often incompetent.

I tend to lose control when I am in a great mood.

I feel completely worthless.

I rarely live up to my own values or standards.

I feel sad.

I feel that I could not shake off the blues even with the help of family and friends.

Section 7 - **Advertisements and Social Media** Please indicate **how often in the last year YOU have seen advertisements** for the following types of gambling.

[For the following questions the choices were “More than 6 times”, “Less than 6 times”, “Never”]

Lottery tickets such as Tattslotto, Powerball, or Keno?

Instant scratch tickets ('scratchies')?

Raffle or fund-raising tickets?

Betting on horse races, trots or dog races?

Betting on sports like football, tennis, rugby or cricket?

Betting on gaming tables at casinos?

Poker machines at casinos?

Poker machines at pubs, hotels or sporting clubs?

Betting on cards or board games with family or friends?

Betting on games of skill such a pool, bowling or darts?

Betting on arcade or video games?

Gambling on the Internet?

Section 7 - **Advertisements and Social Media**

Please indicate **how often in the last year YOU have received promotions** (e.g. a flyer in the mail) for the following types of gambling.

Same list of questions and same scale as above

Section 7 - **Advertisements and Social Media**

Please indicate **how often in the last year YOU have discussed online** (e.g. via email, Facebook, Twitter, Instagram etc.) the following types of gambling.

Same list of questions and same scale as above.

Section 7 - **Advertisements and Social Media**

Please indicate **how often in the last year YOU have discussed offline** (e.g., in person, on the telephone etc.) the following types of gambling.

Same list of questions and same scale as above.

In responding to this survey, did you experience any difficulties or find any of the questions confusing? If so, please explain below. [Free response]
